# Diagnosis of Hand Infections in Intravenous Drug Users by Ultrasound and Water Bath: A Case Series

**DOI:** 10.5811/cpcem.2018.1.36283

**Published:** 2018-03-14

**Authors:** John DeAngelis, Erika St. James, Penelope C. Lema

**Affiliations:** * University at Buffalo Jacobs School of Medicine and Biomedical Sciences, Department of Emergency Medicine, Buffalo, New York; † Cambridge Health Alliance, Department of Emergency Medicine, Cambridge, Massachusetts

## Abstract

We present three cases of hand injury by intravenous drug users in which point-of-care ultrasound, using a specific water bath technique, was able to quickly and efficiently delineate severity of injury. This technique benefited these patients by allowing a painless assessment of their injury for soft tissue injury vs. abscess formation and allowed providers to determine at the bedside whether these patients required immediate surgical intervention.

## INTRODUCTION

It can be difficult to determine the severity of a hand infection. While deep space infections, tenosynovitis, and necrotizing fasciitis of the hand can cause significant morbidity and in some cases mortality if untreated with surgery or intravenous (IV) antibiotics, superficial infections can often be treated with oral antibiotics on an outpatient basis.^1^ The most common cause of acute hand infections is direct inoculation followed by neglect of the wound, such as may be encountered in IV substance abuse.^1^,^2^ Hand infections are often associated with severe pain that can limit patient cooperation and a thorough physical examination.

The use of ultrasound with a water bath is well tolerated and has been described as a technique to improved the resolution of superficial structures.^3^ The recent opioid epidemic in the United States will likely contribute to an increased presentation of soft tissue complications due to IV injections.^4^ Patients at high risk for long-term morbidity secondary to hand infection are repeatedly injecting narcotics into the dorsal hand under unsterile conditions. We present three cases from a county emergency department (ED) in which the water bath ultrasound technique was used to help differentiate superficial injuries from serious hand infections. This technique was performed at the bedside and well tolerated by patients who were unable to tolerate a thorough physical examination due to extreme discomfort or pain.

## CASE REPORT

### Case 1

A 23-year-old female with a history of IV drug use presented to our ED with complaint of a swollen left hand. She had injected heroin into her left hand one week prior. Her exam was notable for a swollen, warm, extremely tender dorsal left hand. The patient’s discomfort with palpation of the hand limited the physical examination. The patient was unable to extend her fingers due to pain.

**Image 1:**
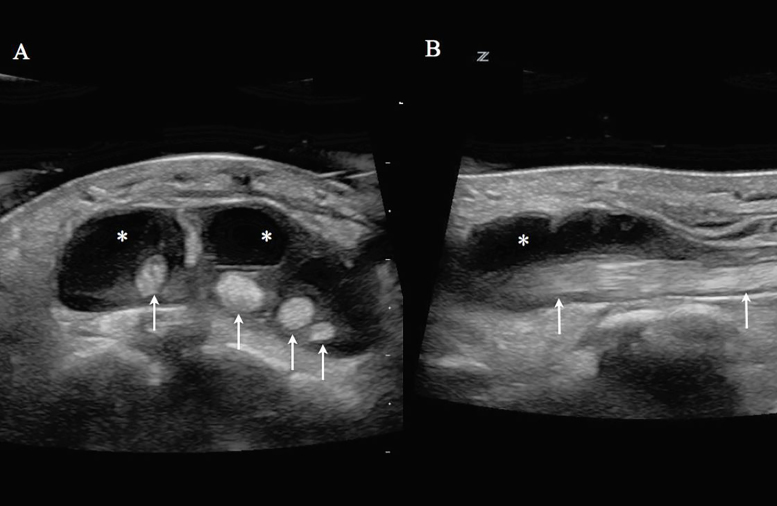
Ultrasound with water bath of a patient with extensor tenosynovitis of the hand due to intravenous drug use. (A) Transverse view of the left hand with fluid collection (asterisk) surrounding the dorsum of the second to fifth digit extensor tendons (arrows). (B) Longitudinal view of the third-digit extensor tendon. Fluid collection (asterisk) surrounding the tendon (arrows).

Her hand was placed in a warm water bath at the bedside. A point-of-care ultrasound (POCUS) evaluation of her hand was performed by an emergency physician (EP) using a Zonare Z.One Ultra 10MHz linear transducer (Mountain View, CA). A large fluid collection was noted along the extensor tendon sheaths of the second to fifth digits with surrounding cobblestoning of the soft tissue (Image 1). These ultrasound findings raised the suspicion of extensor tenosynovitis, and we consulted the orthopedic service. A bedside incision and drainage performed by the orthopedic resident revealed purulent discharge. The patient was emergently sent to the operating room. Intraoperatively, it was confirmed that she had extensor tenosynovitis with dorsal extensor compartment syndrome. The wound culture resulted in Group A β-hemolytic streptococcus.

### Case 2

**Image 2:**
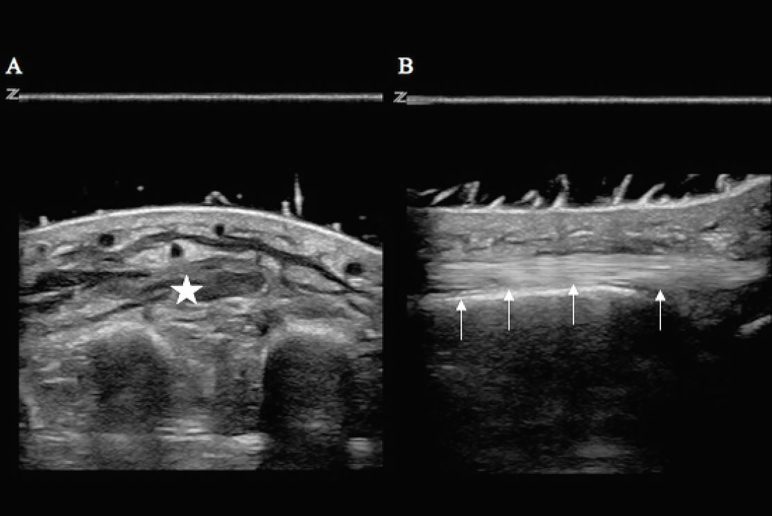
Ultrasound with water bath of a patient’s hand with infiltration of the vein due to intravenous cocaine injection. (A) Transverse view of the right hand with fluid and cobblestoning within the soft tissue (star) without a discrete abscess or fluid collection. (B) Longitudinal view of the right hand extensor tendon (arrows) without a discrete fluid collection.

A 47-year-old female with a history of IV drug use was transferred to our ED with complaint of a swollen right hand. She had injected heroin into her right hand one day prior. Her exam was notable for swelling of the dorsum of her right hand associated with warmth and extreme tenderness with any movement of the digits. The patient had a limited physical examination due to pain. Her hand was placed in a warm water bath at the bedside. An EP then performed a POCUS evaluation of her hand with a Zonare Z.One Ultra 10MHz linear transducer (Mountain View, CA). The ultrasound image revealed diffuse cobblestoning and fluid within the soft tissue with no abscess or discrete fluid collection (Image 2). Orthopedic consult was obtained. The patient was diagnosed with an extravasation injury due to infiltration of the vein and she was discharged home on oral antibiotics.

### CASE 3

A 24-year-old male with a history of IV drug use presented to our ED with complaint of a swollen left hand, fever and chills. The patient had injected cocaine into his left hand three days prior to evaluation. His exam was notable for diffuse swelling and tenderness to the volar and dorsal aspects of his hand. He was in extreme pain, which limited the physical examination.

**Image 3:**
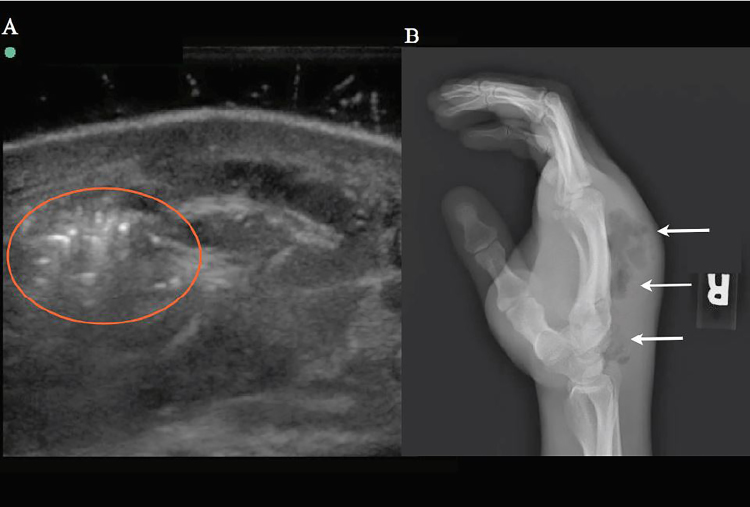
Ultrasound with water bath and radiograph of a patient with necrotizing fasciitis of the hand due to intravenous drug use. (A) Transverse ultrasound view of the right hand with echogenic air (red circle) within the soft tissue. (B) Lateral radiography of the right hand with extensive soft tissue swelling and air within the dorsum of the hand (arrows).

The patient’s hand was placed in a warm water bath at the bedside. A POCUS evaluation of the patient’s hand was performed by an EP with a Sonosite M-Turbo 10MHz linear transducer (Bothell, WA). The ultrasound revealed extensive, soft tissue swelling with small areas of fluid collection and air artifacts concerning for necrotizing fasciitis (Image 3a). The orthopedic service was consulted. Further radiographic evaluation confirmed the ultrasound finding of subcutaneous air (Image 3b). The patient went to the operating room with orthopedics for incision and drainage. Intraoperatively, he was confirmed with necrotizing fasciitis and compartment syndrome of the hand. Blood and wound cultures resulted in *Bacteroides pyogenes* and streptococcal species.

## DISCUSSION

The use of ultrasound to diagnose acute finger or hand infections in the emergency setting has been previously described by Blavais et al.^3^ They also incorporated the use of a water bath to improve image quality of superficial structures. We employed this technique successfully in these three cases to help identify and risk-stratify patients with hand infections related to substance abuse injection. With heroin abuse on the rise in the U.S., healthcare providers will likely see increasing rates of hand infection secondary to IV drug use.^4^ These infections will typically be in the dorsum of the hand due to the availability of venous access in that area. Because the dorsal hand is the most common site of hand infections, non-sterile injections and re-use of needles are likely to result in a prime environment for infections of the hand, deep space, and tendons therein.^2^ These infections often cause exquisite pain and swelling, which can limit examination of the affected area and lead to unidentified abscess or necrotizing fasciitis.

**CPC-EM Capsule**

What do we already know about this clinical entity?

Hand injuries secondary to injection drug use and the water bath technique have both been previously described in the literature.

What makes this presentation of disease reportable?

The combination of the water bath technique with this type of injury is an easy application of ultrasound technology, which directly aids in diagnosis and is readily available in most departments.

What is the major learning point?

Use of point-of-care ultrasound in the emergency department (ED) for prompt diagnosis of hand injuries requiring admission is relatively easy and effective. The use of a water bath helps with patient comfort and may improve image quality.

How might this improve emergency medicine practice?

This technique is easily employed in most EDs and will aid in making treatment plans. It has the potential to become a regular part of emergency practice for injuries of this type.

## CONCLUSION

Ultrasound has been shown to be useful in the diagnosis of necrotizing fasciitis and infections of the hand.^5^,^6^,^7^ The addition of a water bath at bedside is an easy intervention to improve patient comfort and imaging.^3^ In these cases we were able to separate two patients who needed urgent surgical intervention and IV antibiotics from another who had a more superficial extravasation injury and could be discharged home with outpatient therapy. This intervention was quick and well tolerated by the patients. The one patient with superficial injury was able to go home without an unnecessary observational stay, while the case of necrotizing fasciitis was identified almost immediately and brought to the operating room in a very timely fashion. As more patients present to the ED with hand infections related to substance abuse, POCUS with addition of the water bath may be a valuable diagnostic adjunct for rapid differentiation of soft tissue infections of the hand so that they can be appropriately diagnosed and treated in a timely manner.

Documented patient informed consent and/or Institutional Review Board approval has been obtained and filed for publication of this case report.
